# WHEN AND WHY OPERATE ELDERLY OBESE

**DOI:** 10.1590/S0102-6720201500S100022

**Published:** 2015-12

**Authors:** Paulo Afonso Nunes NASSIF, Osvaldo MALAFAIA, Jurandir Marcondes RIBAS-FILHO, Nicolau Gregori CZECZKO, Rodrigo Ferreira GARCIA, Bruno Luiz ARIEDE

**Affiliations:** Post-Graduate Program in Principles of Surgery, Evangelic Faculty of Paraná/University Evangelic Hospital of Curitiba/Medical Research Institute, Curitiba, PR, Brazil

**Keywords:** Obesity, Bariatric surgery, Complications, Elderly

## Abstract

**Introduction:**

: Concurrently with the pandemic obesity is observed global aging phenomenon, with a significant increase of obesity in the elderly population.

**Aim:**

: To review the indications for bariatric surgery for the elderly, mainly focusing on the morbidity and mortality of procedures.

**Method:**

: Review of the literature in PubMed/Medline and Scielo focusing on the relationship of risk factors with different techniques of bariatric surgery in the elderly. The following descriptors were crossed in the form of AND/OR: Obesity; Bariatric surgery; Complications; Elderly.

**Conclusion:**

: In people older than 60 years bariatric procedures represent acceptable and effective treatment option. The elderly should be treated in specialized centers with experience in major surgical procedures and low morbimortality. Going in this way, they experience the benefits of bariatric surgery with acceptable morbidity and mortality. However, age alone should not be considered as an absolute impediment for surgical indication.

## Abstract (pt)

**Introdução:**

Concomitantemente com a pandemia da obesidade, observa-se fenômeno de envelhecimento global, com aumento significativo na população de idosos obesos.

**Objetivo:**

Revisar as indicações da cirurgia bariátrica para os idosos, focando principalmente a morbimortalidade dos procedimentos.

**Método:**

: Revisão da literatura nas bases PubMed/Medline e Scielo com foco na relação dos fatores de risco com diversas técnicas de cirurgia bariátrica em idosos. Foram cruzados os seguintes descritores na forma de AND/OR: Obesidade; Cirurgia bariátrica; Complicações; Idosos.

**Conclusão:**

Nas pessoas com idade superior a 60 anos os procedimentos bariátricos representam opção aceitável e efetiva de tratamento. Os idosos devem ser tratados em centros especializados, de grande movimento cirúrgico e baixos índices de morbidade e mortalidade^13^. Com isso, eles experimentam os benefícios da cirurgia bariátrica com aceitável morbimortalidade. Contudo, a idade por si só não deve funcionar como um impeditivo absoluto na indicação cirúrgica.

## INTRODUCTION

Concurrently with the pandemic obesity, global aging phenomenon is observed with significant increase in obesity on elderly population^5^.

Most countries accepted the chronological age of 65 as the definition of "elderly". At the moment, there is no standard numerical criterion, but the UN agreed that the cut point is 60 or more years to refer older population.

In the US the incidence of elderly obese is increasing; study shows that in the range of 60 to 69 years, 42.5% women and 38.1% of men are obese. Among 70 and 79 years, 31.9% women and 28.9% of men are in this condition^20^. In Brazil, in people over 65 years the prevalence of obesity is 8.7% among men and 16.1% among women^1^^,^ 3 ^,^ 9 ^,^ 15.

The operative bariatric procedures have assumed increasingly important role in the therapeutic arsenal at these ages and have proven to be the most effective treatment in controlling associated co-morbidities. So, this article aims to review the surgical indications on elderly obese, focusing mainly the morbidity and mortality of procedures.

## METHOD

Were selected 21 studies surveyed on the basis of PubMed/Medline and Scielo describing the relationship of risk factors with different techniques of bariatric surgery in the elderly. The following descriptors were crossed in the form of AND/OR: Obesity; Bariatric surgery; complications; Elderly.

## RESULT

With the higher prevalence of obesity, along with increased life expectancy, the number of elderly who need bariatric surgery has also increased. The guidelines of the National Institute of Health - NIH - of the United States of America^12^, when reported that older obese have relative contraindication, the data were based on previous studies showing high morbimortality in this population^16^. National Hospital Discharge Survey and National Inpatient Survey examined about 25,000 bariatric operations and showed mortality of 3.2% in the elderly against 0.2-0.7% in younger, and adverse effects at 32, 3% against 21.6% in the same conditions^2^. Nelson et al.^11^ reported mortality rate of 4% and risk of surgical complications up to 20% in the elderly.

 However, recent articles state that bariatric laparoscopic surgery in patients in this age group is safe and also very effective in remission and control of diseases associated with obesity, improving quality of life, both with Roux-en-Y gastrojejunal bypass, as well as with sleeve gastrectomy^6^^,^ 17.

With increasing experience, confidence in surgical techniques and perioperative care, morbidity and mortality figures lower than it used to be^4^.

Old age in obesity leads to changes in body composition - in particular its visceral component - and is associated with increased incidence of sarcopenia, defined as a syndrome characterized by progressive and widespread loss of muscle mass and strength, increased risk of nutritional deficiencies, worst quality and reduced life expectation^18^^,^ 19.

The surgical indications in older obese is equal to that for adults, according to guidelines issued by the World Health Organization, ie IMC≥40 or IMC≥35 with comorbidities. In this particular group must be applied more rigorous verification of satisfactory clinical and surgical conditions, due to lower physiological reserve and tolerance for complications. The aim of the operation in the elderly is to increase disability-free survival, improve quality of life and comorbidities control.

Interference in some functional activity is more related with BMI than with age. Based on this observation, the greater the BMI greater the benefit for the elderly after the operation; it should be focused on high BMI instead of age at time of surgery^15^.

Despite studies showed mortality is higher in patients over 65 years, evidence shows that between 60 and 65 can be performed the operation with mortality equal to the youngest. Over 65 years, yes, it should be evaluated clinically even more carefully - preferably for more quantitative than qualitative criteria - verifying the actual risk/benefit of the operation^13^.

The best performance with the operative techniques and perioperative management led to decreased morbidity and mortality. The Roux-en-Y gastrojejunal bypass is the most performed procedure, providing loss of significant and sustained weight in the long run. Several researchers published their good results regarding safety and efficacy in elderly^6^^,^ 7 ^,^ 10.

The sleeve gastrectomy has evolved considerably since its initial introduction as a preliminary operation, and today, as final operation in high-risk patients. It takes advantage for being a technique that maintains the duodenum transit, which has beneficial effect for elderly patients, who do not have the drawbacks of malabsorption that occurs in bypass^8^.

Clinical improvement of comorbidities, reducing the use of drugs and improving quality of life are significant results after bariatric surgery in older with successful weight loss^14^.

## CONCLUSION

In people older than 60 years bariatric procedures represent acceptable and effective treatment option. The elderly should be treated in specialized centers with experience in major surgical procedures and low morbimortality. Going in this way, they experience the benefits of bariatric surgery with acceptable morbidity and mortality. However, age alone should not be considered as an absolute impediment for surgical indication.
